# Supplementary material for: Microglial dopamine receptor elimination defines sex-specific nucleus accumbens development and social behavior in adolescent rats
Source: Nat Commun. 2018 Sep 25;9:3769. doi: 10.1038/s41467-018-06118-z (PMC6156594; doi:10.1038/s41467-018-06118-z)
Supplement: Supplementary file 1 — Supplementary Information [file 41467_2018_6118_MOESM1_ESM.pdf]

# Microglial dopamine receptor elimination defines sex-specific nucleus accumbens development and social behavior in adolescent rats

Ashley M. Kopec, Caroline J. Smith, Nathan R. Ayre, Sean C. Sweat, Staci D. Bilbo

**Supplementary Materials**

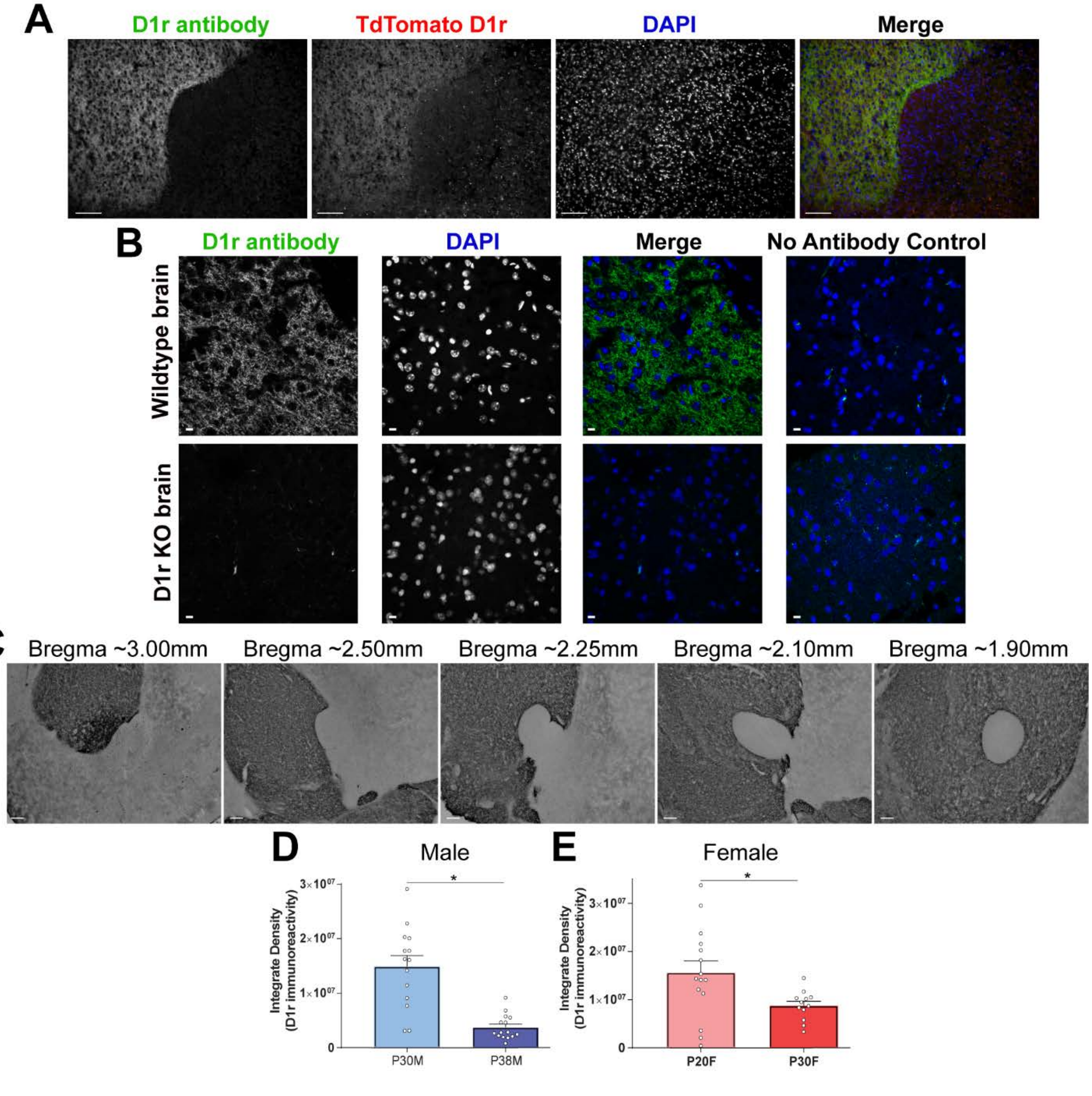


**Supplementary Fig. 1. D1r immunoreactivity in the dorsomedial NAc**

**(A)** Examining the spatial patterns of overlap in the NAc between anti-D1r antibody immunoreactivity in D1r-Tdtomato mouse brain. Scale bar equals 100µm. (B) Examining high magnification immunoreactivity of anti-D1r antibody in wildtype and D1r knock out (KO) mice. No anti-D1r antibody immunoreacitivty was observed in any of 6 different brain sections spanning the anterior-posterior axis of the D1r-rich dorsal striatum and NAc. Scale bar equals 20µm. **(C)** Chromogenic immunohistochemistry of D1r reactivity in the NAc (example images are male). In all images, the midline is toward the left and dorsal

brain is toward the top. In immunofluorescent analyses (Fig. 1), z-stacks were acquired in the dorso-medial D1r enriched areas of the NAc. Scale bar equals 100µm. **(D)** D1r downregulation between P30-38 in males (Supplementary Table 1A) and **(E)** P20-30 in females (Supplementary Table 1B) was confirmed (Fig. 1) in a separate cohort (*n*=3 animals/sex/age). Data were analyzed with two-tailed unpaired *t*-tests. Histograms portray the mean +/- SEM with individual data points overlaid. Significant unpaired *t*-test ($p<0.05$) comparisons are delineated with an asterisk. All statistics are in Table S1.

| | Comparison | Statistical test | *n* | Statistic | *p* -value | Outliers? | Figure |
|---|---|---|---|---|---|---|---|
| A | D1r: P30-38 Males | unpaired *t* -test | 3 animals; 8 sections/animal | $t_{(27)}$=5.56 | <0.001 | N/A | Supp. Fig. 1D |
| B | D1r: P20-30 Females | unpaired t-test | 3 animals; 8 sections/animal | $t_{(25)}$=2.41 | 0.024 | N/A | Supp. Fig. 1E |

**Supplementary Table 1. Detailed Statistics**

Statistical details for every analysis in Supplementary Fig. 1.

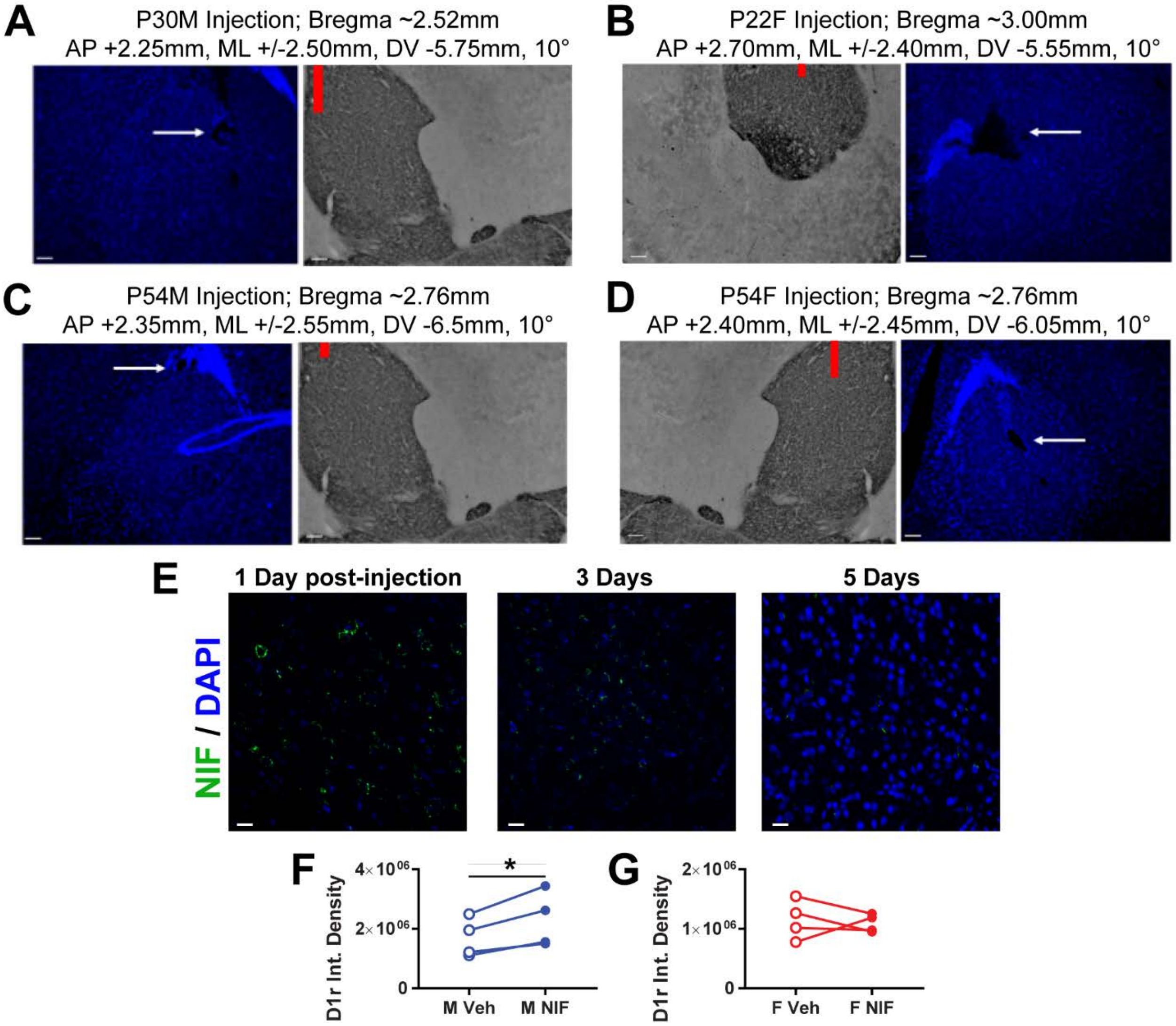


**Supplementary Fig. 2. Microinjection coordinates and *in vivo* NIF manipulations**

Stereotaxic coordinates and volumes were estimated by injecting bromophenol blue dye into the brain under high levels of anesthesia (non-survival), immediately sacrificing the animal, and taking coronal sections through the brain. The microinjection technique was selected for these experiments to reduce the level of inflammatory (i.e. microglial) reaction to the manipulation, as we hypothesized there was an on-going, natural immune process occurring in adolescent development. Brain healing was so effective that microinjection track marks were undetectable in the vast majority of experimental animals (8 days after injection). Because we are uncertain that the dye used to estimate coordinates under very high anesthesia would recapitulate dynamics of NIF/vehicle injection under lower anesthesia (survival surgeries), we microinjected NIF bilaterally in males and females, allowed the animals to wake and resume normal behavior, and then sacrificed the animals 2-4 hours later. Example microinjection tracts for a **(A)** P30 male, **(B)** P22 female, **(C)** P54 male, and **(D)** P54 female are visualized with DAPI to help detect neural architecture and comparable locations from previously collected D1r

immunohistochemistry (see Supplementary Fig. 1). In both sexes, the tract targets the dorso-medial NAc. **(E)** To examine how long after injection NIF remains detectable in the NAc, we analyzed tissue 1, 3, and 5 days after unilateral injection. NIF immunoreactivity (via 6xHis tag IHC) was detectable in the NAc for 3-5 days post-injection. Scale bar equals 50 µm. **(F-G)** Raw densitometric values of D1r immunoreactivity from the experiment presented in Fig. 3G-H. Data were analyzed with two-tailed paired *t*-tests. Histograms portray the individual data points from each hemisphere. Significant two-tailed paired *t*-tests ($p$<0.05) comparisons are delineated with an asterisk. All statistics are in Supplementary Table 2.

| | Comparison | Statistical test | *n* | Statistic | *p* -value | Outliers? | Figure |
|---|---|---|---|---|---|---|---|
| A | *In vivo* matched NIF-Vehicle injection raw D1r densitometry: Male | paired *t* -test | 4 animals; 4-7 sections/animal | $t_{(3)}$=4.14 | 0.026 | N/A | Supp. Fig. 2F |
| B | *In vivo* matched NIF-Vehicle injection raw D1r densitometry: Female | paired t-test | 4 animals; 4-7 sections/animal | $t_{(3)}$=0.75 | 0.745 | N/A | Supp. Fig. 2G |

**Supplementary Table 2. Detailed Statistics**

Statistical details for every analysis in Supplementary Fig. 2.

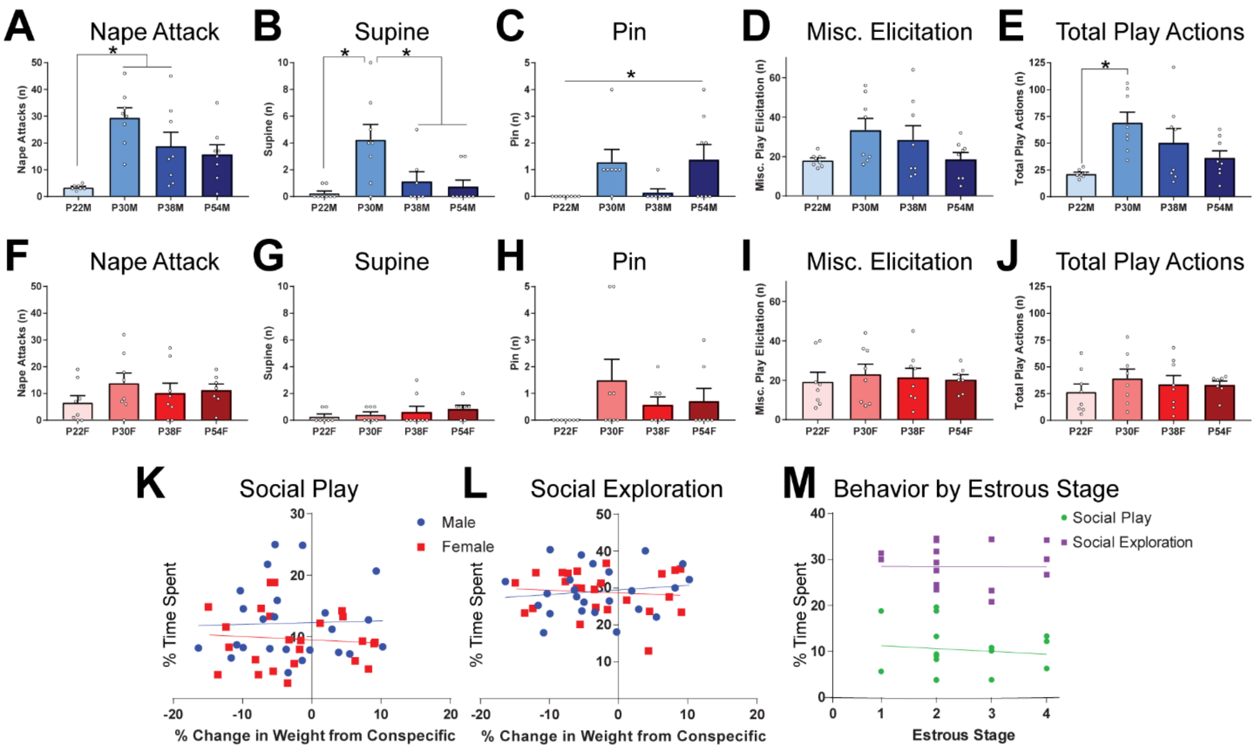


**Supplementary Fig. 3. Components of social play in normally developing adolescent male and female rats**

For all groups, nape attacks, supine positions, pin positions, and miscellaneous play elicitations were counted in social behavior test videos (*n*=8/sex/age). Total play actions is the sum of all these components. Males: **(A)** Nape attacks (Supplementary Table 3A), **(B)** supine positions (Supplementary Table 3B), and **(E)** total play actions (Supplementary Table 3E) were highest at P30, an age at which total play is also highest (Fig. 4). **(C)** There was a significant difference among groups in number of pins, but no individual comparisons reached statistical threshold (Supplementary Table 3C), and there was no change in **(D)** misc. elicitation (Supplementary Table 3D). Females: **(E-J)** There was no change in any individual component of play over development (Supplementary Table 3F-J). Behavioral data were analyzed with one-way ANOVAs and Holm-Sidak's post-hoc comparisons. Change in weight between the experimental and conspecific animals was calculated as a percent change from conspecific (Experimental-Conspecific / Experimental * 100), and then plotted against total play or social exploration scores. **(K)** Pearson correlations between weight and total play were nonsignificant for males (Supplementary Table 3K) and females (Supplementary Table 3L). **(L)** Pearson correlations between weight and social exploration were nonsignificant for males (Supplementary Table 3M) and females (Supplementary Table 3N). **(M)** If experimental females had vaginal openings (only 1 female at P30 and all females at P38 and P54), estrous smears were collected and classified as metestrus, diestrus, proestrus, or estrus via light microscopy. Total play and social exploration behavior scores were then plotted against these four categories. Pearson correlations between estrous phase and total play (Supplementary Table 3O) and social exploration (Supplementary Table 3P) were nonsignificant. Histograms portray the mean ± SEM

with individual data points overlaid. Significant post-hoc Holm-Sidak *t*-test (A-J) and Pearson's *r* (K-M) ($p<0.05$) comparisons are delineated with an asterisk. All statistics are in Supplementary Table 3.

| | Comparison | Statistical test | *n* | Statistic | *p* -value | Outliers? | Figure |
|---|---|---|---|---|---|---|---|
| A | Social Play across development: Nape attacks; Males | one-way ANOVA | 8 animals/age | $F_{(3,27)}$=7.78 | <0.001 | P20: 1 | Supp. Fig. 3A |
| | P22:P30 | Holm-Sidak's posthoc | 7:8 | $t_{(27)}$=4.79 | <0.001 | | |
| | P22:P38 | Holm-Sidak's posthoc | 7:8 | $t_{(27)}$=2.84 | 0.042 | | |
| | P22:P54 | Holm-Sidak's posthoc | 7:8 | $t_{(27)}$=2.27 | 0.092 | | |
| | P30:P38 | Holm-Sidak's posthoc | 8:8 | $t_{(27)}$=2.02 | 0.103 | | |
| | P30:P54 | Holm-Sidak's posthoc | 8:8 | $t_{(27)}$=2.62 | 0.056 | | |
| | P38:P54 | Holm-Sidak's posthoc | 8:8 | $t_{(27)}$=0.59 | 0.557 | | |
| B | Social Play across development: Supine; Males | one-way ANOVA | 8 animals/age | $F_{(3,27)}$=6.54 | 0.002 | P38: 1 | Supp. Fig. 3B |
| | P22:P30 | Holm-Sidak's posthoc | 8:8 | $t_{(27)}$=4.79 | 0.003 | | |
| | P22:P38 | Holm-Sidak's posthoc | 8:7 | $t_{(27)}$=2.84 | 0.778 | | |
| | P22:P54 | Holm-Sidak's posthoc | 8:8 | $t_{(27)}$=2.27 | 0.856 | | |
| | P30:P38 | Holm-Sidak's posthoc | 8:7 | $t_{(27)}$=2.02 | 0.022 | | |
| | P30:P54 | Holm-Sidak's posthoc | 8:8 | $t_{(27)}$=2.62 | 0.008 | | |
| | P38:P54 | Holm-Sidak's posthoc | 7:8 | $t_{(27)}$=0.59 | 0.856 | | |
| C | Social Play across development: Pin; Males | one-way ANOVA | 8 animals/age | $F_{(3,26)}$=3.74 | 0.023 | P30: 1; P38: 1 | Supp. Fig. 3C |
| | P22:P30 | Holm-Sidak's posthoc | 8:7 | $t_{(26)}$=2.39 | 0.117 | | |
| | P22:P38 | Holm-Sidak's posthoc | 8:7 | $t_{(26)}$=0.27 | 0.957 | | |
| | P22:P54 | Holm-Sidak's posthoc | 8:8 | $t_{(26)}$=2.64 | 0.080 | | |
| | P30:P38 | Holm-Sidak's posthoc | 7:7 | $t_{(26)}$=2.05 | 0.143 | | |
| | P30:P54 | Holm-Sidak's posthoc | 7:8 | $t_{(26)}$=0.17 | 0.957 | | |
| | P38:P54 | Holm-Sidak's posthoc | 7:8 | $t_{(26)}$=2.29 | 0.117 | | |
| D | Social Play across development: Misc. Elicitation; Males | one-way ANOVA | 8 animals/age | $F_{(3,28)}$=2.32 | 0.097 | N/A | Supp. Fig. 3D |
| E | Social Play across development: Total Play Actions; Males | one-way ANOVA | 8 animals/age | $F_{(3,27)}$=4.87 | 0.008 | P22: 1 | Supp. Fig. 3E |
| | P22:P30 | Holm-Sidak's posthoc | 7:8 | $t_{(27)}$=3.64 | 0.007 | | |
| | P22:P38 | Holm-Sidak's posthoc | 7:8 | $t_{(27)}$=2.21 | 0.136 | | |
| | P22:P54 | Holm-Sidak's posthoc | 7:8 | $t_{(27)}$=1.15 | 0.456 | | |
| | P30:P38 | Holm-Sidak's posthoc | 8:8 | $t_{(27)}$=1.48 | 0.385 | | |
| | P30:P54 | Holm-Sidak's posthoc | 8:8 | $t_{(27)}$=2.59 | 0.075 | | |
| | P38:P54 | Holm-Sidak's posthoc | 8:8 | $t_{(27)}$=1.10 | 0.456 | | |
| F | Social Play across development: Nape attacks; Females | one-way ANOVA | 8 animals/age | $F_{(3,27)}$=0.91 | 0.448 | P54: 1 | Supp. Fig. 3F |
| G | Social Play across development: Supine; Females | one-way ANOVA | 8 animals/age | $F_{(3,25)}$=0.68 | 0.572 | P22: 1, P30: 1; P54: 1 | Supp. Fig. 3G |
| H | Social Play across development: Pin; Females | one-way ANOVA | 8 animals/age | $F_{(3,25)}$=1.53 | 0.232 | P22: 1, P30: 1; P54: 1 | Supp. Fig. 3H |
| I | Social Play across development: Misc. Elicitation; Females | one-way ANOVA | 8 animals/age | $F_{(3,27)}$=0.13 | 0.940 | P54: 1 | Supp. Fig. 3I |
| J | Social Play across development: Total Play Actions; Females | one-way ANOVA | 8 animals/age | $F_{(3,27)}$=0.53 | 0.665 | P54: 1 | Supp. Fig. 3J |
| K | Social Play across development by weight: Males | Pearson's *r* | 8 animals/age | *r* =0.03 | 0.874 | N/A | Supp. Fig. 3K |
| L | Social Play across development by weight: Females | Pearson's *r* | 8 animals/age | *r* =-0.09 | 0.691 | N/A | Supp. Fig. 3K |
| M | Social Exploration across development by weight: Males | Pearson's *r* | 8 animals/age | *r* =0.14 | 0.528 | N/A | Supp. Fig. 3L |
| N | Social Exploration across development by weight: Females | Pearson's *r* | 8 animals/age | *r* =-0.09 | 0.670 | N/A | Supp. Fig. 3L |
| O | Social Play across development by Estrous stage | Pearson's *r* | 8 animals/age | *r* =-0.11 | 0.670 | N/A | Supp. Fig. 3M |
| P | Social Exploration across development by Estrous Stage | Pearson's *r* | 8 animals/age | *r* =-0.004 | 0.988 | N/A | Supp. Fig. 3M |

**Supplementary Table 3. Detailed Statistics**

Statistical details for every analysis in Supplementary Fig. 3.

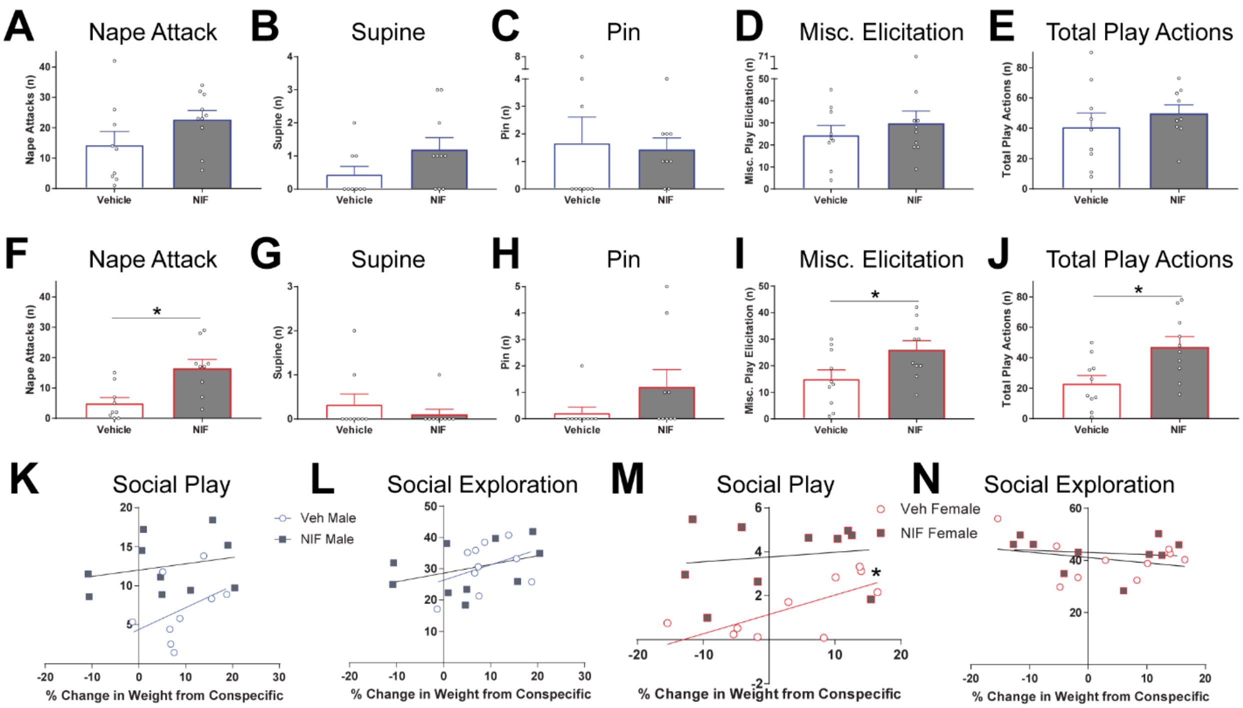


**Supplementary Fig. 4. Components of social play in P38 males and P30 females following bilateral vehicle/NIF injection**

For all groups, nape attacks, supine positions, pin positions, and miscellaneous play elicitations were counted in social behavior test videos (*n*=10/sex/age). Total play actions is the sum of all these components. Males: **(A-E)** There was no significant change in any individual component of play by NIF (Supplementary Table 4A-E). Females: **(F)** Nape attacks (Supplementary Table 4F), **(I)** misc. elicitation (Supplementary Table 4I), and **(J)** total play actions (Supplementary Table 4J) were higher in NIF-treated females. There was no significant change in **(G)** supine (Supplementary Table 4G) or **(H)** pin positions (Supplementary Table 4H). Behavioral data were analyzed with 2-tailed unpaired *t*-tests. Change in weight between the experimental and conspecific animals was calculated as a percent change from conspecific (Experimental-Conspecific / Experimental * 100), and then plotted against total play or social exploration scores. **(K)** Pearson correlations between weight and total play were nonsignificant for vehicle- and NIF-treated males (Supplementary Table 4K,L). **(L)** Pearson correlations between weight and social exploration were nonsignificant for vehicle- and NIF-treated males (Supplementary Table 4M,N). **(M)** Pearson correlations between weight and total play were significant for vehicle-treated (Supplementary Table 4O) and nonsignificant for NIF-treated females (Supplementary Table 4P). **(N)** Pearson correlations between weight and social exploration were nonsignificant for vehicle- and NIF-treated females (Supplementary Table 4Q,R). Histograms portray the mean ± SEM with individual data points overlaid. Significant unpaired *t*-test (A-J) and Pearson's *r* (K-N) ($p$<0.05) comparisons are delineated with an asterisk. All statistics are in Supplementary Table 4.

| | Comparison | Statistical test | *n* | Statistic | *p* -value | Outliers? | Figure |
|---|---|---|---|---|---|---|---|
| A | Social Play Vehicle:NIF treated Males: Nape attacks | unpaired *t* -test | 10 animals/treatment | $t_{(17)}$=1.61 | 0.125 | Veh: 1 | Supp. Fig. 4A |
| B | Social Play Vehicle:NIF treated Males: Supine | unpaired *t* -test | 10 animals/treatment | $t_{(17)}$=1.71 | 0.107 | Veh: 1 | Supp. Fig. 4B |
| C | Social Play Vehicle:NIF treated Males: Pin | unpaired *t* -test | 10 animals/treatment | $t_{(16)}$=0.22 | 0.832 | Veh: 1; NIF: 1 | Supp. Fig. 4C |
| D | Social Play Vehicle:NIF treated Males: Misc. Elicitation | unpaired *t* -test | 10 animals/treatment | $t_{(17)}$=0.77 | 0.453 | Veh: 1 | Supp. Fig. 4D |
| E | Social Play Vehicle:NIF treated Males: Total Play Actions | unpaired *t* -test | 10 animals/treatment | $t_{(16)}$=0.84 | 0.414 | Veh: 1; NIF: 1 | Supp. Fig. 4E |
| F | Social Play Vehicle:NIF treated Females: Nape attacks | unpaired *t* -test | 10 animals/treatment | $t_{(16)}$=3.39 | 0.004 | Veh: 1; NIF: 1 | Supp. Fig. 4F |
| G | Social Play Vehicle:NIF treated Females: Supine | unpaired *t* -test | 10 animals/treatment | $t_{(16)}$=0.85 | 0.406 | Veh: 1; NIF: 1 | Supp. Fig. 4G |
| H | Social Play Vehicle:NIF treated Females: Pin | unpaired *t* -test | 10 animals/treatment | $t_{(16)}$=1.48 | 0.160 | Veh: 1; NIF: 1 | Supp. Fig. 4H |
| I | Social Play Vehicle:NIF treated Females: Misc. Elicitation | unpaired *t* -test | 10 animals/treatment | $t_{(18)}$=2.34 | 0.031 | N/A | Supp. Fig. 4I |
| J | Social Play Vehicle:NIF treated Females: Total Play Actions | unpaired *t* -test | 10 animals/treatment | $t_{(18)}$=2.86 | 0.010 | N/A | Supp. Fig. 4J |
| K | Social Play Vehicle:NIF treatment by weight: Vehicle Males | Pearson's *r* | 10 animals/treatment | $r$=0.41 | 0.271 | N/A | Supp. Fig. 4K |
| L | Social Play Vehicle:NIF treatment by weight: NIF Males | Pearson's *r* | 10 animals/treatment | $r$=0.25 | 0.487 | N/A | Supp. Fig. 4K |
| M | Social Exploration Vehicle:NIF treatment by weight: Vehicle Males | Pearson's *r* | 10 animals/treatment | $r$=0.37 | 0.290 | N/A | Supp. Fig. 4L |
| N | Social Exploration Vehicle:NIF treatment by weight: NIF Males | Pearson's *r* | 10 animals/treatment | $r$=0.37 | 0.288 | N/A | Supp. Fig. 4L |
| O | Social Play Vehicle:NIF treatment by weight: Vehicle Females | Pearson's *r* | 10 animals/treatment | $r$=0.71 | 0.022 | Veh: 1 | Supp. Fig. 4M |
| P | Social Play Vehicle:NIF treatment by weight: NIF Females | Pearson's *r* | 10 animals/treatment | $r$=0.15 | 0.678 | N/A | Supp. Fig. 4M |
| Q | Social Exploration Vehicle:NIF treatment by weight: Vehicle Females | Pearson's *r* | 10 animals/treatment | $r$=-0.29 | 0.425 | N/A | Supp. Fig. 4N |
| R | Social Exploration Vehicle:NIF treatment by weight: NIF Females | Pearson's *r* | 10 animals/treatment | $r$=-0.14 | 0.709 | N/A | Supp. Fig. 4N |

**Supplementary Table 4. Detailed Statistics**

Statistical details for every analysis in Supplementary Fig. 4.

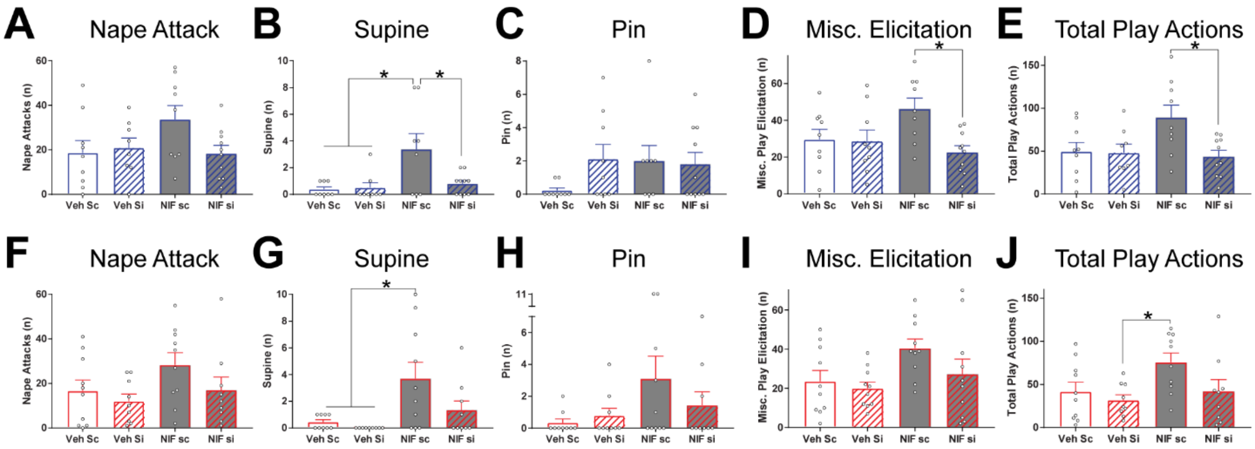


**Supplementary Fig. 5. Components of social play in P38 males and P30 females following bilateral vehicle/NIF + D1r siRNA/scRNA co-injection**

For all groups, nape attacks, supine positions, pin positions, and miscellaneous play elicitations were counted in social behavior test videos (*n*=9-10/sex/age). Total play actions is the sum of all these components. Males: **(B)** Supine positions (Supplementary Table 5B), **(D)** misc. elicitation (Supplementary Table 5D), and **(E)** total play actions (Supplementary Table 5E) were highest in NIF + scRNA treated males. There was no significant change in **(A)** nape attacks (Supplementary Table 5A) or **(C)** pin positions (Supplementary Table 5C). Females: **(G)** Supine positions (Supplementary Table 5G) and **(J)** total play actions (Supplementary Table 5J) were highest in NIF + scRNA treated females. There was no significant change in **(F)** nape attacks (Supplementary Table 5F), **(H)** pins (Supplementary Table 5H), or **(I)** misc. elicitation (Supplementary Table 5I). Behavioral data were analyzed with one-way ANOVAs and Holm-Sidak's post-hoc comparisons. Histograms portray the mean ± SEM with individual data points overlaid. Significant post-hoc Holm-Sidak *t*-test ($p$<0.05) comparisons are delineated with an asterisk. All statistics are in Supplementary Table 5.

| | Comparison | Statistical test | *n* | Statistic | *p* -value | Outliers? | Figure |
|---|---|---|---|---|---|---|---|
| A | Social Play: NIF/Vehicle + si/scRNA Nape Attacks; Males | one-way ANOVA | 9-10 animals/treatment | $F_{(3,32)}$=2.10 | 0.120 | Veh si: 1 | Supp. Fig. 6A |
| B | Social Play: NIF/Vehicle + si/scRNA Supine; Males | one-way ANOVA | 9-10 animals/treatment | $F_{(3,30)}$=5.22 | 0.005 | Veh sc: 1; Veh si: 1; NIF sc: 1 | Supp. Fig. 6B |
| | P22:P30 | Holm-Sidak's posthoc | 8:8 | $t_{(30)}$=0.14 | 0.943 | | |
| | P22:P38 | Holm-Sidak's posthoc | 8:8 | $t_{(30)}$=3.40 | 0.011 | | |
| | P22:P54 | Holm-Sidak's posthoc | 8:10 | $t_{(30)}$=0.51 | 0.943 | | |
| | P30:P38 | Holm-Sidak's posthoc | 8:8 | $t_{(30)}$=3.26 | 0.014 | | |
| | P30:P54 | Holm-Sidak's posthoc | 8:10 | $t_{(30)}$=0.36 | 0.943 | | |
| | P38:P54 | Holm-Sidak's posthoc | 8:10 | $t_{(30)}$=3.08 | 0.018 | | |
| C | Social Play: NIF/Vehicle + si/scRNA Pin; Males | one-way ANOVA | 9-10 animals/treatment | $F_{(3,32)}$=1.49 | 0.237 | NIF sc: 1 | Supp. Fig. 6C |
| D | Social Play: NIF/Vehicle + si/scRNA Misc. Elicitation; Males | one-way ANOVA | 9-10 animals/treatment | $F_{(3,33)}$=3.64 | 0.023 | N/A | Supp. Fig. 6D |
| | P22:P30 | Holm-Sidak's posthoc | 9:9 | $t_{(33)}$=0.12 | 0.908 | | |
| | P22:P38 | Holm-Sidak's posthoc | 9:9 | $t_{(33)}$=2.19 | 0.136 | | |
| | P22:P54 | Holm-Sidak's posthoc | 9:10 | $t_{(33)}$=0.94 | 0.732 | | |
| | P30:P38 | Holm-Sidak's posthoc | 9:9 | $t_{(33)}$=2.31 | 0.130 | | |
| | P30:P54 | Holm-Sidak's posthoc | 9:10 | $t_{(33)}$=0.82 | 0.732 | | |
| | P38:P54 | Holm-Sidak's posthoc | 9:10 | $t_{(33)}$=3.18 | 0.019 | | |
| E | Social Play: NIF/Vehicle + si/scRNA Total Play Actions; Males | one-way ANOVA | 9-10 animals/treatment | $F_{(3,32)}$=3.84 | 0.019 | Veh si: 1 | Supp. Fig. 6E |
| | P22:P30 | Holm-Sidak's posthoc | 9:8 | $t_{(32)}$=0.10 | 0.971 | | |
| | P22:P38 | Holm-Sidak's posthoc | 9:9 | $t_{(32)}$=2.58 | 0.068 | | |
| | P22:P54 | Holm-Sidak's posthoc | 9:10 | $t_{(32)}$=0.40 | 0.971 | | |
| | P30:P38 | Holm-Sidak's posthoc | 8:9 | $t_{(32)}$=2.60 | 0.068 | | |
| | P30:P54 | Holm-Sidak's posthoc | 8:10 | $t_{(32)}$=0.28 | 0.671 | | |
| | P38:P54 | Holm-Sidak's posthoc | 9:10 | $t_{(32)}$=3.04 | 0.028 | | |
| F | Social Play: NIF/Vehicle + si/scRNA Nape Attacks; Females | one-way ANOVA | 10 animals/treatment | $F_{(3,34)}$=2.00 | 0.133 | Veh si: 1; NIF si: 1 | Supp. Fig. 6F |
| G | Social Play: NIF/Vehicle + si/scRNA Supine; Females | one-way ANOVA | 10 animals/treatment | $F_{(3,33)}$=5.15 | 0.005 | Veh sc: 1; Veh si: 1; NIF si: 1 | Supp. Fig. 6G |
| | P22:P30 | Holm-Sidak's posthoc | 9:9 | $t_{(33)}$=0.42 | 0.679 | | |
| | P22:P38 | Holm-Sidak's posthoc | 9:10 | $t_{(33)}$=3.14 | 0.018 | | |
| | P22:P54 | Holm-Sidak's posthoc | 9:9 | $t_{(33)}$=0.83 | 0.652 | | |
| | P30:P38 | Holm-Sidak's posthoc | 9:10 | $t_{(33)}$=3.57 | 0.007 | | |
| | P30:P54 | Holm-Sidak's posthoc | 9:9 | $t_{(33)}$=1.25 | 0.524 | | |
| | P38:P54 | Holm-Sidak's posthoc | 10:9 | $t_{(33)}$=2.28 | 0.117 | | |
| H | Social Play: NIF/Vehicle + si/scRNA Pin; Females | one-way ANOVA | 10 animals/treatment | $F_{(3,33)}$=1.87 | 0.155 | Veh sc: 1; Veh si: 1; NIF si: 1 | Supp. Fig. 6H |
| I | Social Play: NIF/Vehicle + si/scRNA Misc. Elicitation; Females | one-way ANOVA | 10 animals/treatment | $F_{(3,36)}$=2.63 | 0.065 | N/A | Supp. Fig. 6I |
| J | Social Play: NIF/Vehicle + si/scRNA Total Play Actions; Females | one-way ANOVA | 10 animals/treatment | $F_{(3,34)}$=3.34 | 0.030 | Veh si: 1; NIF si: 1 | Supp. Fig. 6J |
| | P22:P30 | Holm-Sidak's posthoc | 10:9 | $t_{(34)}$=0.67 | 0.874 | | |
| | P22:P38 | Holm-Sidak's posthoc | 10:10 | $t_{(34)}$=2.32 | 0.125 | | |
| | P22:P54 | Holm-Sidak's posthoc | 10:9 | $t_{(34)}$=0.03 | 0.973 | | |
| | P30:P38 | Holm-Sidak's posthoc | 9:10 | $t_{(34)}$=2.93 | 0.036 | | |
| | P30:P54 | Holm-Sidak's posthoc | 9:9 | $t_{(34)}$=0.68 | 0.874 | | |
| | P38:P54 | Holm-Sidak's posthoc | 10:9 | $t_{(34)}$=2.22 | 0.125 | | |

**Supplementary Table 5. Detailed Statistics**

Statistical details for every analysis in Supplementary Fig. 5.

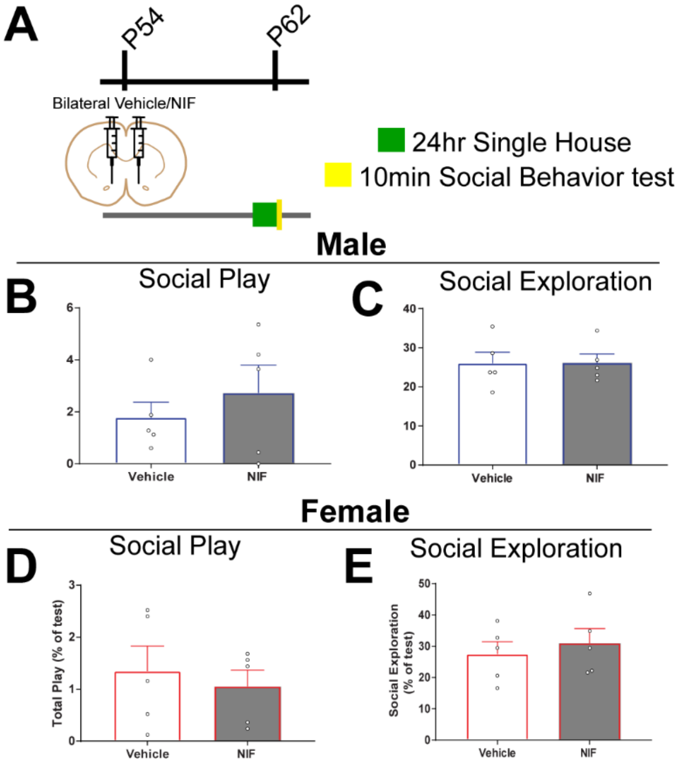


**Supplementary Fig. 6. NIF manipulation of the NAc in P54 males and females does not change social behaviors.**

**(A)** Experimental design: Animals were bilaterally injected with vehicle or NIF at P54, single-housed for 24hrs at P61, and then assessed for social behaviors at P62 (*n*=5/sex/treatment). Male **(B)** social play (Supplementary Table 6A) and **(C)** social exploration (Supplementary Table 6B) was not changed by NIF. Female **(D)** social play (Supplementary Table 6C) and **(E)** social exploration (Supplementary Table 6D) was not changed by NIF. Data were analyzed with 2-tailed unpaired *t*-tests. Histograms portray the mean ± SEM with individual data points overlaid. Significant unpaired *t*-test ($p$<0.05) comparisons are delineated with an asterisk. All statistics are in Supplementary Table 6.

| | Comparison | Statistical test | *n* | Statistic | *p* -value | Outliers? | Figure |
|---|---|---|---|---|---|---|---|
| A | Social Play Vehicle:NIF treated P54 Males | unpaired *t* -test | 5 animals/treatment | $t_{(8)}$=0.78 | 0.457 | N/A | Supp. Fig. 5B |
| B | Social Exploration Vehicle:NIF treated P54 Males | unpaired *t* -test | 5 animals/treatment | $t_{(8)}$=0.04 | 0.969 | N/A | Supp. Fig. 5C |
| C | Social Play Vehicle:NIF treated P54 Females | unpaired *t* -test | 5 animals/treatment | $t_{(8)}$=0.50 | 0.631 | N/A | Supp. Fig. 5D |
| D | Social Exploration Vehicle:NIF treated P54 Females | unpaired *t* -test | 5 animals/treatment | $t_{(8)}$=0.57 | 0.585 | N/A | Supp. Fig. 5E |

**Supplementary Table 6. Detailed Statistics**

Statistical details for every analysis in Supplementary Fig. 6.
